# Healthcare Programmes for Truck Drivers in Sub-Saharan Africa: A Systematic Review and Meta-Analysis

**DOI:** 10.1371/journal.pone.0156975

**Published:** 2016-06-22

**Authors:** Samanta Tresha Lalla-Edward, Siyabulela Christopher Fobosi, Catherine Hankins, Kelsey Case, W. D. Francois Venter, Gabriela Gomez

**Affiliations:** 1 Wits Reproductive Health and HIV Institute, University of the Witwatersrand, Johannesburg, South Africa; 2 Department of Global Health/Amsterdam Institute for Global Health and Development, Academic Medical Centre, University of Amsterdam, Amsterdam, The Netherlands; 3 Department of Infectious Disease Epidemiology, London School of Hygiene and Tropical Medicine, London, United Kingdom; 4 Department of Infectious Disease Epidemiology, Imperial College London, London, United Kingdom; 5 Department of Global Health, London School of Hygiene and Tropical Medicine, London, United Kingdom; Simon Fraser University, CANADA

## Abstract

**Background:**

Truck drivers have unique health needs, and by virtue of their continuous travel, experience difficulty in accessing healthcare. Currently, planning for effective care is hindered by lack of knowledge about their health needs and about the impact of on-going programmes on this population’s health outcomes. We reviewed healthcare programmes implemented for sub-Saharan African truck drivers, assessed the evaluation methods, and examined impact on health outcomes.

**Methods:**

We searched scientific and institutional databases, and online search engines to include all publications describing a healthcare programme in sub-Saharan Africa where the main clients were truck drivers. We consulted experts and organisations working with mobile populations to identify unpublished reports. Forest plots of impact and outcome indicators with unadjusted risk ratios and 95% confidence intervals were created to map the impact of these programmes. We performed a subgroup analysis by type of indicator using a random-effects model to assess between-study heterogeneity. We conducted a sensitivity analysis to examine both the summary effect estimate chosen (risk difference vs. risk ratio) and model to summarise results (fixed vs. random effects).

**Results:**

Thirty-seven publications describing 22 healthcare programmes across 30 countries were included from 5,599 unique records. All programmes had an HIV-prevention focus with only three expanding their services to cover conditions other primary healthcare services. Twelve programmes were evaluated and most evaluations assessed changes in input, output, and outcome indicators. Absence of comparison groups, preventing attribution of the effect observed to the programme and lack of biologically confirmed outcomes were the main limitations. Four programmes estimated a quantitative change in HIV prevalence or reported STI incidence, with mixed results, and one provided anecdotal evidence of changes in AIDS-related mortality and social norms. Most programmes showed positive changes in risk behaviours, knowledge, and attitudes. Our conclusions were robust in sensitivity analyses.

**Conclusion:**

Diverse healthcare programmes tailored to the needs of truck drivers implemented in 30 sub-Saharan African countries have shown potential benefits. However, information gaps about availability of services and their effects impede further planning and implementation of effective healthcare programmes for truck drivers.

## Introduction

Transport workers, such as truck drivers, have specific healthcare needs. Globally, they bear a disproportionate health burden, including high rates of sexually transmitted infections (STI), cancer, cardiovascular diseases, chronic conditions (predominantly diabetes, obesity, backache, leg pains), respiratory diseases, and an array of mental health conditions (with the most common being depression, anxiety, chronic insomnia, personality disorders and post-traumatic stress disorder). Occupational factors that increase risk include irregular schedules, sedentary lifestyle due to long hours of driving/sitting, musculoskeletal and other injuries due to loading and unloading cargo, exposure to road accidents and deaths, extended periods of social isolation, unhealthy food choices on the road and poor access to healthcare [1–7].

In sub-Saharan Africa, transport corridors are essential for local economies due to lack of waterways and inadequate rail services. These corridors have been characterised as being affected by transience, unemployment, and poverty [8–10]. Because of the transcontinental nature of the transport industry, health programmes prioritising truck drivers require complementary national healthcare policies. Most countries in the region are aware of the susceptibility of transport workers to poor health outcomes and Ministries of Transport and Health have begun to develop strategic plans to address this issue [11]. However, public sector financial and human resources constraints have delayed progress [12].

The trucking industry in sub-Saharan Africa is predominantly privately-run and has attracted international donor and domestic funding for work-related programmes tackling different aspects of truck drivers’ health needs. Increasingly, implementation has been proceeding across the region of healthcare programmes prioritising truck drivers and concentrating on services to increase awareness and identification of communicable (STI, HIV (including prevention of mother to child transmission (PMTCT), tuberculosis, and malaria) and chronic diseases (hypertension and diabetes), as well as general primary healthcare [13–36]. Nevertheless, further planning for efficient implementation, scale-up, and sustainability of healthcare programmes for truck drivers is hindered by knowledge gaps about this population’s needs and the impact of existing healthcare services on health outcomes. In this review, our objective is to describe healthcare programmes implemented in sub-Saharan Africa prioritising truck drivers, assess the methods used to evaluate them, and evaluate their impact on truck drivers’ health outcomes.

## Methods

We performed a systematic review of the published and unpublished literature following the registered protocol (CRD42014013327) on the international prospective register of systematic reviews, Prospero [37]. We adhered to the PRISMA guidelines for reporting of systematic reviews and meta-analyses; the PRISMA checklist is provided in S1 Table [38].

### Search strategy and study selection

We searched scientific databases such as PubMed/Medline and ISI Web of Knowledge, institutional databases (University of Witwatersrand, Imperial College London, London School of Hygiene and Tropical Medicine, University of North Carolina), non-profit organisations and country-level reports (South African National AIDS Council, USAID-PEPFAR, NGO websites), and online search engines (google, google scholar) using a broad search strategy including both MeSH headings and free text (29 permutations of search terms related to: 1) the population (truck driver, lorry driver, long haul driver, long distance driver, driver); 2) intervention (health intervention); and 3) outcome (odds ratio, risk ratio, evaluation, cost-effectiveness, effect, impact, effectiveness)), with no date or language limitations. Experts and non-profit organisations working with truck drivers (or with migrant populations in general) were consulted separately to identify unpublished reports. We also reviewed country-level reports of funders and NGO (i.e. South African National AIDS Council, USAID-PEPFAR, NGO websites). Citations and bibliographies of records were reviewed to identify additional relevant material. All searches were run independently by two researchers (STLE and SCF). Results were downloaded, duplicates removed, and a database of all possible records organised for review by the end of January 2015.

Titles and abstracts were then screened by two independent reviewers (STLE and GG) to include all publications and reports describing a healthcare programme in sub-Saharan Africa providing services designed specifically for truck drivers. The full text records of all the material selected for further examination were assessed independently for inclusion. Two reviewers (STLE and GG) compared the final list of selected material.

### Data extraction and analysis

We designed a pre-defined tool to extract data describing each programme, its evaluation method and reported results. Data included: implementation and evaluation years, implementation and evaluation locations, services provided, service providers, funders, evaluators, details of evaluation method, and reported results. One reviewer undertook the data extraction (STLE) and a second reviewer (GG) conducted a quality control check.

We present tabulated data describing current and past healthcare programmes, providing an overview of these programmes in the region by type and coverage over time. We then provide a critical assessment and a narrative review of the evaluation methods used. Methodological quality of each evaluation was assessed on the basis of study’s internal validity (sites and population included, sample size, and sampling method), data reported, and whether a comparison group was included in the analysis. Due to variation across studies in the ways of reporting evaluation results, all indicators reported were mapped against a logic model for programme evaluation connecting the following elements: inputs (e.g., staff), activities (e.g., trainings, services), outputs (e.g., clients served, tests conducted), and results ranging from intermediate outcomes (e.g., risk behaviour change) to long-term impact (e.g., reduction in STI incidence) [39].

Whenever possible, we calculated unadjusted risk ratios (RRs) and 95% confidence intervals (CIs) from data provided. We present these RRs and 95% Cl for all impact and outcome indicator results in forest plots to provide an overview of the potential impact these programmes have had on truck driver health. We performed a sensitivity analysis to assess the robustness of our findings to the choice of summary statistic and calculated unadjusted risk differences and 95% CI from data provided as an alternative. Because the results presented in the forest plots presented significant heterogeneity, we assessed the sources of between-study heterogeneity through a subgroup analysis by type of indicator. Outcome indicators were assessed in two broad categories: 1) indicators of risk behaviour (condom use, number of sexual partners, alcohol and drug use) and 2) indicators measuring knowledge, attitudes, and perceptions. Impact indicators included reported measures of HIV prevalence and STI incidence. We applied a random-effects model to calculate summary RRs and 95% Cl by subgroup. The robustness of our findings to this methodological decision was tested by re-running the analysis using a fixed-effects model to calculate summary statistics by subgroup. All analyses were conducted in STATA 12.

### Ethics statement

The proposed study was approved by the University of the Witwatersrand Human Research Ethics Committee (M140506) as one of the objectives in a process evaluation project of North Star Alliance’s services in Southern Africa.

## Results

**Fig 1:**
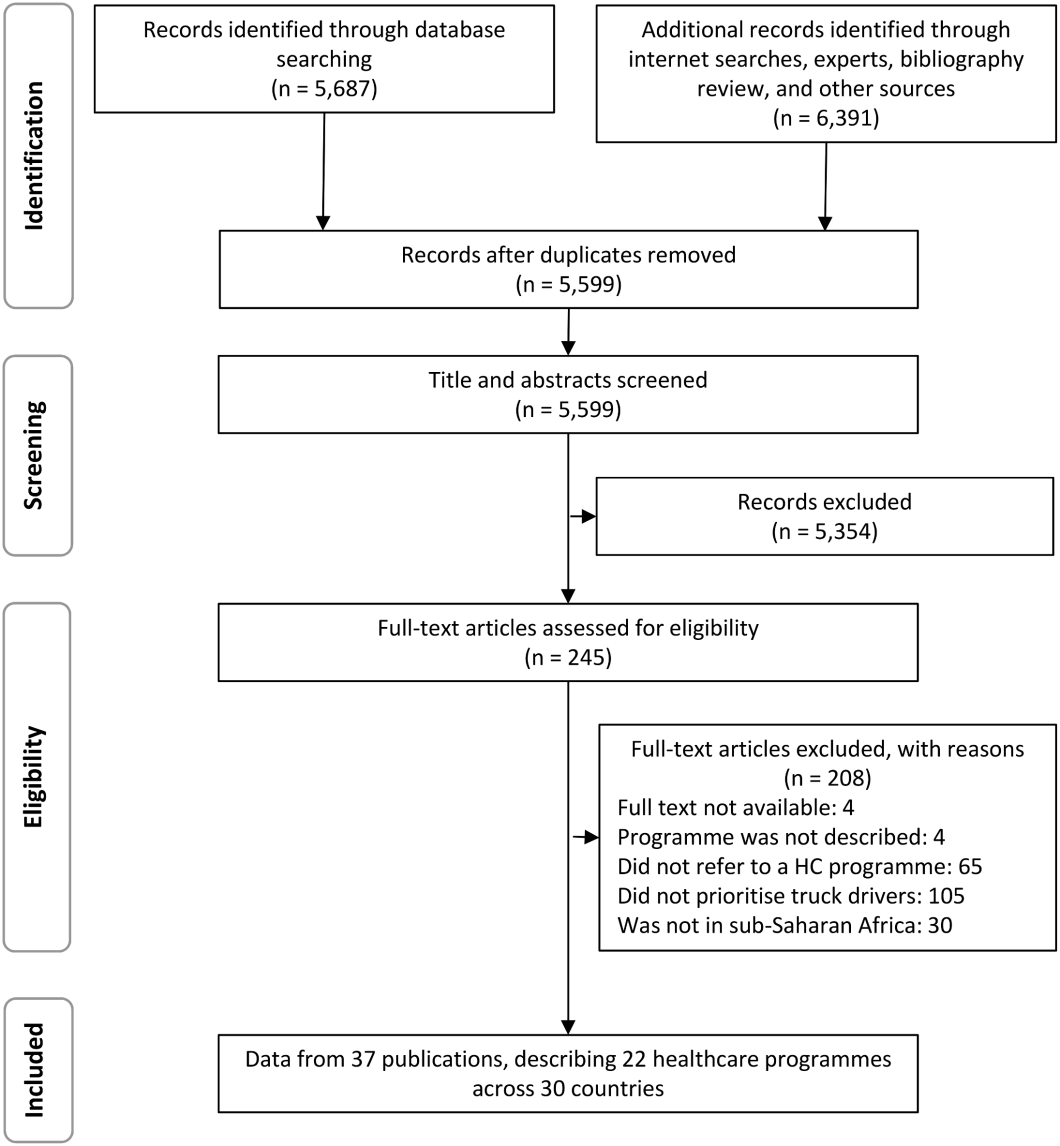
Flow diagram of study selection. n, number; HC, healthcare.

We included 37 publications from 5,599 identified unique records. The results of the searches and selection process are summarised in Fig 1.

### Overview of healthcare programmes for truck drivers

The selected publications described 22 healthcare programmes across 30 countries. Table 1 presents a summary description of each programme.

**Table 1:** Description of healthcare programmes for truck drivers in sub-Saharan Africa.

Programme	Duration	Countries	Description	Funded by	Eval.
AMREF Truck Driver project [26, 45]	1989–1993	TZA	Implementers: PE. Clients: TD, FSW. Interventions: IEC	USAID/AMREF	Yes
World Vision [30]	1990- current	MOZ, SWZ, ZMB, ZWE	Implementers: PE, HCW. Clients: TD, FSW, OVC. Interventions: IEC, PMTCT, income generating activities	WFP, SADC	No
Prevention of sexual transmission of HIV through IEC [25]	1991–1994	MWI	Implementers: PE. Clients: TD, FSW. Intervention: IEC	European Commission	Yes
NECTOI's AIDS education program [22, 23]	1992–1997	ZWE	Implementers: PE. Clients: TD, FSW. Intervention: IEC, including drama, radio programmes	NECTOI, SIDA, USAID, FHI—AIDSCAP, NDA	Yes
Behavioural risk-reduction programme [21]	1993	KEN	Implementers: Mobile team*. Clients: TD. Intervention: IEC, HTC, condom distribution, STI testing and treatment	US NIH	Yes
The Nigeria STD control intervention [24]	1996–1997	NGA	Implementers: State University Ado-Ekiti. Clients: TD, SW, SW managers, students. Intervention: IEC, stigma reduction, condom distribution and demonstration	SIDA	No
Prévention du SIDA sur les Axes Migratoire de l’Afrique de l’Ouest (PSAMAO) [29]	1997–2000	BEN, BFA, CMR, CIV, MLI, NER, TGO	Implementers: PE. Clients: TD, FSW, seasonal migrant workers. Intervention: IEC, mass media, condom demonstration, inter-educational cassettes	USAID	Yes
Chevron Workplace AIDS Prevention Programme [28]	1998-current	NGA	Implementers: HCW. Clients: TD. Intervention: IEC, poetry, drama, music video shows, moonlight storytelling, condom promotion, positive living, STI treatment, HTC, PMTCT	Chevron	No
Corridor Empower Project/Trucking Wellness [33]	1999-current	ZAF	Implementers: HCW, PE. Clients: TD. Intervention: STI treatment, HTC, IEC, condom distribution, PC, ART, nutrition supplements	PPP	No
Corridors of Hope [27, 40–43]	1999-current	DRC, ZMB	Implementers: HCW, PE. Clients: TD, FSW. Intervention: HTC, condom distribution, STI and malaria screening and treatment, FP, TB screening and referral, participatory learning, economic strengthening activities	USAID, JICA	Yes
	1999-current	ZMB	Expanded clients to out-of-school youth (10–24yrs)	USAID	Yes
	1999-current	ANG, BWA, LSO, MWI, MOZ, NAM, ZAF, SWZ, ZMB, ZWE	Expanded clients to traders	USAID	No
Reproductive Health services for populations at high risk of HIV in Mozambique [34]	2001	MOZ	Implementers: night-clinic HCW, PE. Clients: TD, SW. Intervention: STI testing and treatment, IEC, condom distribution, outreach, FP, HTC	FICA	Yes
Ugandan Transport Sector Workplace HIV Interventions [30, 44]	2001–2003	UGA	Implementers: HCW, PE**. Clients: TD, transport employees. IEC, condom distribution, forming CBOs at truck stops	UCTOA, ITF, AGTWU-URWU, Ugandan MoH	No
High Risk Corridors Initiative (HRCI) [30, 50]	2001–2008	DJI, ETH	Implementers: PE, HBC volunteers, HCW, spiritual counsellors. Clients: TD, transport workers, FSW, youth in and out of school, influential leaders, civil servants, OVC, PLWHA. Intervention: IEC, HTC, ART, STI screening, OI treatment/referral, livelihood enhancement	USAID	Yes
ADRA's HIV/AIDS Programme [31]	2002–2005	GHA	Implementers: PE. Clients: TD, mechanics, tailors, hairdressers. Intervention: drama activities, posters, radio talk shows, durbars	USAID	Yes
The T-MARC Project [35, 61]	2004–2010	TZA	Implementers (Formative phase): FHI360. Clients: counsellors and NGO workers Implementers (Intervention phase): counsellors and NGO workers. Clients: TD, SW, tan boys and miners. Interventions: IEC (including “being faithful”), male and female condoms, FP, ORS.	USAID	No
WFP's HIV/AIDS training [30]	2004- current	BDI, DRC, DJI, ETH, ERI, KEN, RWA, SLE, TZA, UGA	Implementers: trainers. Clients: transport and contract workers. Intervention: IEC, stigma and discrimination reduction messages	WFP	No
HIV/AIDS project for the Abidjan-Lagos Corridor*** [32, 53]	2004-current	BEN, GHA, CIV, NGA, TGO	Implementers: HCW, PE. Clients: transport workers, migrants, SW, local populations. Intervention: IEC, social marketing of condoms, ART, HTC, STI and OI treatment, HIV grants to CSOs	World Bank (post 2007: Global Fund, USAID)	Yes
GDC Haulage workplace HIV/AIDS Programme [30]	2006	ZWE	Implementers: PE. Clients: TD. Intervention: outreach, video drama activities	GDC Haulage	No
ROADS II Project**** [13–20]	2006	DJI	Implementers: PE. Clients: TD, their assistants, SW, low income women, youth out of school, community men, bar/restaurant employees. Intervention: HTC, BCC, drama, film screening, stigma/GBV reduction, local CBO capacity building	USAID/FHI360	Yes
	2008	RWA	Expanded DJI model to OVC, PLWHA, fishermen, and homeless. Add’l interventions: ART referrals, OVC support, economic opportunities, FP, MCH, nutrition	USAID/FHI360	Yes
	2008	TZA	Expanded RWA model to MSM, IDU. Add’l interventions: condom promotion, IDU, palliative care, HS strengthening	USAID/FHI360	Yes
	2008	UGA	Expanded RWA model to uniformed services, people engaging in MCP. Add’l interventions: condom promotion	USAID/FHI360	Yes
	2009	ZMB	Expanded UGA model to discordant couples. Add’l interventions: male circumcision, screening for TB/malaria, STI syndromic tx, FP	USAID/FHI360	Yes
	2009	BDI	Expanded TZA/ZMB models. Add’l interventions: IEC, PMTCT	USAID/FHI360	Yes
	2010	MOZ	Expanded TZA model. Add’l interventions: wellness clinics	USAID/FHI360, PPP	Yes
	2012	KEN	Expanded DJI model to married youth, older orphans. Add’l interventions: PC, referral	USAID/FHI360	Yes
North Star Alliance [36]	2007-current	BWA, DRC, KEN, MWI, MOZ, ZAF, SWZ, TZA, GMB, UGA, ZMB, ZWE	Implementers: HCW, PE. Clients: TD, their assistants, SW, local community. Interventions: HTC, BCC, condom promotion and distribution, STI testing and syndromic treatment, TB and malaria pre-screening, ART, general check-ups and referral	PPP	No
RTI International’s HIV Prevention Interventions for Most-at-Risk Populations project [47]	2008–2013	BWA	Implementers: PE. Clients: TD, SW, SW clients, young women (15–29 yrs). Intervention: condom promotion and distribution; support of referral; HCW and PE capacity building	USAID	No
Improving HIV/AIDS knowledge and risk behaviours of drivers [48]	2011	NGA	Implementers: PE. Clients: TD. Intervention: three-day intervention (2–3 hours per day) on IEC	n/a	Yes

Notes:

* Mobile team consisting of a physician, nurses, health educator, clerical assistant, driver.

** Not listed but implicit from intervention service delivery package

*** Currently named “Organisation du Corridor Abidjan-Lagos (OCAL) project”;

**** Limited accessible programme information on ROADS I. ROADS I was implemented in south Sudan, Ethiopia, Democratic Republic of Congo and ROADS II countries. Countries: ANG, Angola; BEN, Benin; BWA, Botswana; BFA, Burkina Faso; BDI, Burundi; CMR, Cameroon; DRC, Democratic Republic of Congo; CIV, Côte d'Ivoire; DJI, Djibouti; ERI, Eritrea; ETH, Ethiopia; GHA, Ghana; KEN, Kenya; LSO, Lesotho; MWI, Malawi; MLI, Mali; MOZ, Mozambique; NAM, Namibia; NER, Niger; NGA, Nigeria; RWA, Rwanda; SLE, Sierra Leone; ZAF, South Africa; SWZ, Swaziland; TZA, Tanzania; GMB, The Gambia; TGO, Togo; UGA, Uganda; ZMB, Zambia; ZWE, Zimbabwe.

Abbreviations: Eval, evaluated; PE, peer educator; HCW, healthcare worker; TD, truck driver; FSW, female sex worker; IEC, information, education and communication; USAID, United States Agency for International Development; AMREF, African Medical & Research Foundation; OVC, Orphans and Vulnerable Children; HIV, human immunodeficiency virus; PMTCT, prevention of mother to child transmission; WFP, World Food Program; SADC, Southern African Development Community; NECTOI, National Employment Council for the Transport Operating Industry; AIDS, acquired immunodeficiency syndrome; SIDA, Swedish International Development Agency; FHI (FHI360), Family Health International; NGO, non-governmental organisation; AIDSCAP, AIDS Control and Prevention Project; NDA, Norwegian Development Agency; HTC, HIV testing and counselling; STI, sexually transmitted infection; US NIH, United States National Institutes of Health; ART, antiretroviral treatment; PPP, public-private partnership; FP, family planning; TB, tuberculosis; yrs, years; JICA, Japan International Cooperation Agency; FICA, Flemish International Cooperation Agency; UCTOA, Uganda Commercial Truck Owners Association; ITF, International Transport Forum; AGTWU-URWU, Amalgamated Transport and General Workers' Union- Uganda Railway Workers Union; ADRA, Adventist Development and Relief Agency; CBOs, community-based organisations; MoH, ministry of health; PLWHA, people living with HIV/AIDS; NGO, non-governmental organisation; ORS, oral rehydration solution; OI, opportunistic infections; CSO, community service officers; GBV, gender-based violence; MCH, maternal and child health; BCC, behaviour change communication; MSM, men who have sex with other men; IDU, injection drug users; MCP, multiple and concurrent partnerships; PC, primary care; Add’l, additional; HS, health system.

Although we searched for healthcare programmes in general and aimed to include any programme providing services for any health-related issues, all programmes identified cover HIV-related interventions, with only three programmes expanding their services to cover malaria, tuberculosis, or general primary care services (i.e. ROADS II, Corridors of Hope, and North Star Alliance) [17, 18, 27, 36, 40–43]. The majority of the programmes rely strongly on peer educators and healthcare workers to offer HIV prevention services. These include behaviour change communication (BCC) (n = 15); condom marketing and distribution (n = 16); STI screening and/or syndromic treatment (n = 11); stigma reduction activities (n = 10); information, education, and communication (IEC) (n = 9); HIV testing and counselling (HTC) (n = 9) through outreach or site-based programmes, in addition to capacity building for community or local staff, PMTCT, family planning, income generation activities, and linkage to clinical and social support services in 11 programmes.

The African Medical and Research Foundation (AMREF) implemented the first healthcare programme for truck drivers in the region in 1989 in Tanzania [26]. This programme lasted four years and focused on IEC for HIV prevention. Shortly thereafter, in 1990, World Vision introduced a programme in Southern Africa (Mozambique, Swaziland, Zambia, and Zimbabwe) [30]. The World Vision programme is ongoing, making it the longest running truck driver healthcare programme in sub-Saharan Africa. Fourteen [21–26, 28, 30, 31, 33–35, 44–48] of the 22 identified programmes were delivered within a single country setting, whilst four [13–20, 27, 30, 36, 40–43] programmes have broader geographical coverage, covering eight or more countries.

**Fig 2:**
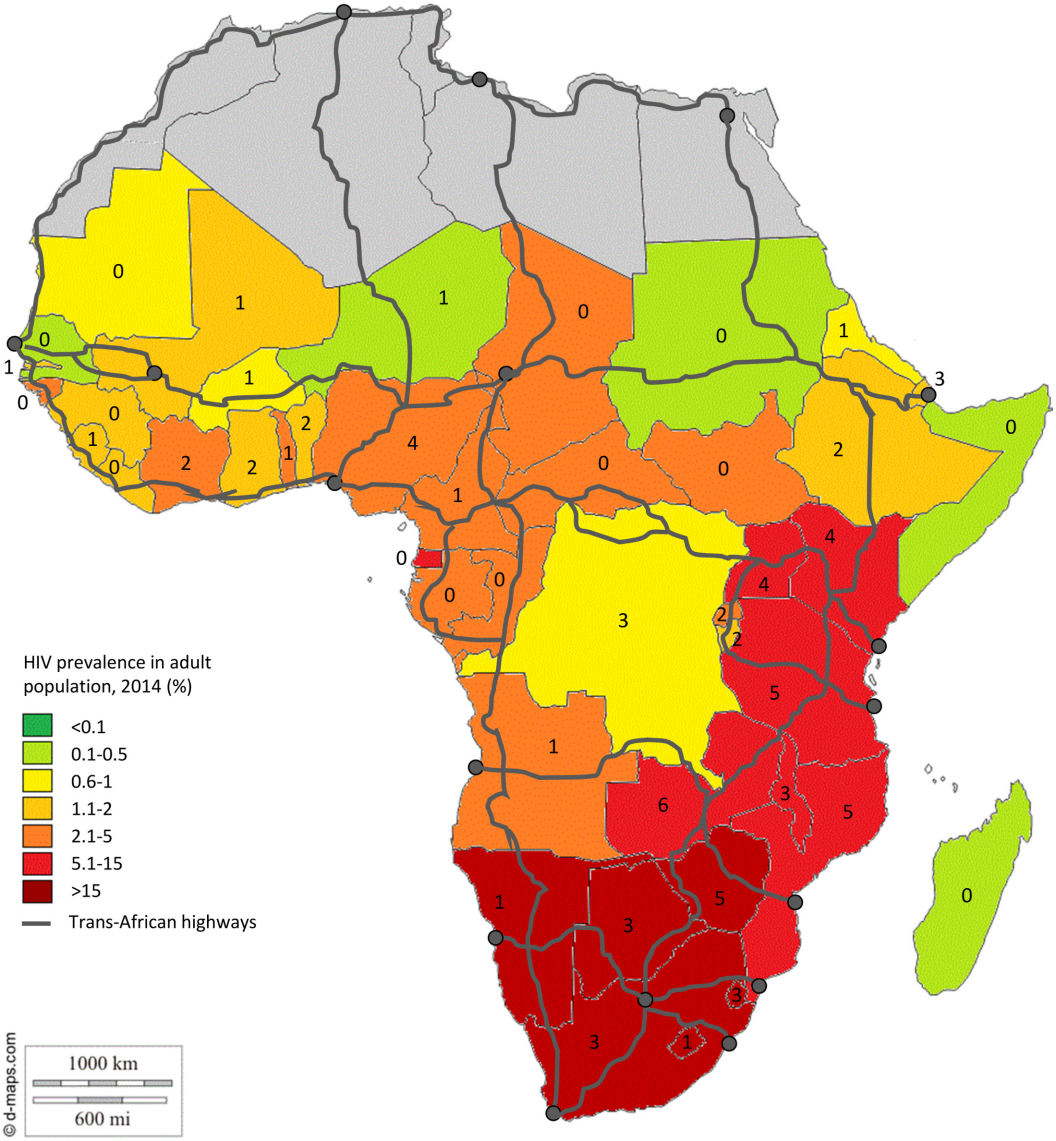
Country-level HIV prevalence and number of healthcare programmes for truck drivers [62]. The numbers shown per country represent the total number of healthcare programmes available in each country for truck drivers.

Programmes with a broad geographical coverage vary in their objectives and approach. The Corridors of Hope project, implemented in 11 countries, provides services to key populations (including truck drivers, female sex workers, and members of surrounding communities) along transport corridors and at border sites. The main focus of the project is comprehensive HIV prevention services and improved linkages and referral networks [43]. The ROADS II project, operational in eight countries, aims primarily to address HIV and other health issues in the communities around transport corridors by engaging with the communities and community leadership to ensure uptake of services provided at the programme’s SafeTstops [13]. The World Food Program’s HIV training programme, implemented in ten countries, is a train-the-trainer initiative for truck drivers and contract workers with a focus on BCC and activities to reduce stigma and discrimination [30]. The North Star Alliance Programme, active in 12 countries, is a primary healthcare programme delivered through stationary and mobile clinics operated by healthcare workers and peer educators [36]. Countries in Southern Africa with high HIV prevalence, such as Zambia, Zimbabwe, Tanzania, and Mozambique, had five or more programmes active nationally. Fig 2 illustrates the distribution of programmes per country against adult HIV prevalence in the general population and along main transport corridors in 2014 [49]. We observed a variety of funders, with domestic investments constituting a minority. Among international funders, USAID represents the largest investor, with just under half of the programmes (n = 9) funded or co-funded by this agency.

### Evaluations of healthcare programmes for truck drivers

Of the 22 programmes identified, just over half (n = 12) were evaluated in 17 countries [21–23, 25–27, 29–32, 34, 40–43, 45, 48–52]. A detailed description of the evaluation methods is provided in Table 2.

**Table 2:** Description of methods used during programme evaluations.

Programme	Year	Country	Sites	Population	Sample size	Sampling	Method and limitations
AMREF Truck Driver project [26, 45]	1990, 1991, 1993	TZA	Seven major truck stops and three trucking companies sites	TD	1990, n = 425 1991, n = 198 1993, n = 305	Random	Internal evaluation. Cross-sectional surveys (KAP) at three time points: baseline, 18 m, 24 m Limitations: No HIV testing, reported prevalence of STI, lack of comparison group
				FSW	1990, n = 304 1991, n = 121 1993, n = 318	Two-stage cluster	
Behavioural risk-reduction programme [21]	1993–1994	KEN	Depots of six trucking companies	Men, HIV-negative, employed at trucking companies	n = 556	All eligible	Internal evaluation. Prospective cohort study, follow up visits every 3m for 12 m. Limitations: Selective population. N = 133 screened but not enrolled; N = 215 lost to follow-up. Symptomatic STI evaluation only, lack of comparison group
NECTOI's AIDS education programme [22, 23]	1995, 1997	ZWE	21 sites on major highways, border crossings, and at the edges of large cities	Transport workers**	N/A	N/A	Internal evaluation. Cross-sectional surveys (KAP) at two times points: baseline, 24 m. Limitations: lack of comparison group, lack of biological data on testing and limited questions on behaviour
				FSW	N/A	N/A	
Prevention of sexual transmission of HIV through IEC[25]	1996	MWI	All Blantyre’s trucking companies	TD	n = 347, one focus group	Random	Internal evaluation. Mixed methods (focus groups, questionnaires). Three groups: 1) active PE, 2) no PE, 3) average (no PE, HCW visits). Limitations: Poor PE retention for follow up assessment, social desirability bias***
			Six districts from two regions	FSW	n = 424, six focus groups	All eligible, bar-based	
Prévention du SIDA sur les Axes Migratoire de l’Afrique de l’Ouest (PSAMAO) [29]	1997, 2000	BFA	One site	TD	1997, n = 831 2000,n = 1032	Two-stage random	Internal evaluation. Cross-sectional surveys (KAP) at two time points: baseline, 36 m. Limitations: lack of comparison group, unique site
Corridors of Hope (CoH) [27, 40–43]	2000	ZMB	Three sites	TD	n = 867	All eligible	External evaluation. Cross-sectional BSS. Limitations: lack of comparison group, lack of biological data on testing.
	2003	ZMB	Two sites	TD	n = 686	All eligible	External evaluation. Cross-sectional BSS. Limitations: lack of comparison group, lack of biological data on testing.
				UP	n = 349		
				M+Light TD	n = 228		
	2005	ZMB	One site	TD	n = 146	All eligible	External evaluation. Cross-sectional BSS. Limitations: unique site, lack of comparison group, lack of biological data on testing.
				UP	n = 206		
				M+Light TD	n = 150		
	2006	ZMB	Three sites	TD	n = 1,006	All eligible (2 sites), cluster (1 site)	Internal evaluation. Cross-sectional BSS. Trend analysis on behaviour indicators (2000, 2003 and 2006). Limitations: lack of comparison group, lack of biological data on testing.
	2008–2009	ZMB	Four sites	TD	n = 1,863	Time-location cluster	Internal evaluation. Cross-sectional BSS. Trend analysis on behaviour indicators (2000, 2003, 2006 and 2009). Limitations: lack of comparison group, lack of biological data on testing.
The HIV/AIDS project for the Abidjan-Lagos Corridor* [32, 53]	2005	BEN, GHA, CIV, NGA, TGO	Eight border crossing points	TD	2005, n = 594 2007, n = 533	N/A	External evaluation. Three population-based surveys (KAP + behaviour + reported STI and HIV prevalence). Limitations: lack of comparison group. Lack of biological data on STI testing.
	2007			FSW	2005, n = 235 2007, n = 188	N/A	
ADRA's HIV/AIDS Programme [31]	2006	GHA	One municipality	TD	n = 5	Stratified random	External evaluation. Cross-sectional survey (questionnaire, discussion checklist). Limitations: lack of comparison group, small sample including PE
				Mechanics	n = 68		
				Hairdressers	n = 61		
				Tailors/DM	n = 66		
High Risk Corridor Initiative (HRCI) [30, 50]	2007	ETH	Six sites	TD, HCW, PE, PLWHA, community†	n = 291	N/A	External evaluation. Mixed method (focus groups, interviews, analysis of routinely-collected data). Limitations: lack of comparison group. Small sample. Routinely collected data not disaggregated by population reached, results presented only in narrative.
Reproductive Health services for populations at high risk of HIV in Mozambique [34]	2007–2009	MOZ	One site	TD	n = 32	Purposive	External evaluation. Mixed method (focus groups, structured interviews, analysis of routinely-collected data, costs). Limitations: lack of comparison group, unique site, small sample
				FSW	n = 16		
				KI	n = 28		
Improving HIV/AIDS knowledge and risk behaviours of drivers [48]	2011	NGA	One site	TD	n = 140 (each group)	Multi-staged	Internal evaluation. Cross-sectional surveys (before and after). Included a control group. Limitations: unique site
ROADS II [51]	2012	BDI, DJI, DRC, KEN, MOZ, RWA, sSUD,TZA, UGA, ZMB	Five sites (Burundi, Kenya, Mozambique, Rwanda, and Tanzania) across three types of programming	TD	n = 20	Purposive	External evaluation. Mixed methods (document review, key information interviews, analysis of routinely-collected data, costs). Limitations: lack of baseline, lack of comparison group, cannot determine impact– 1) qualitative evaluation of visited sites; 2) conducted a year prior to project end
				Other	n = 433		

Notes:

* Currently named “Organisation du Corridor Abidjan-Lagos (OCAL) project”;

** transport workers include truck drivers, bus drivers and their assistants;

*** difference in reported condom usage data between interviews and focus groups;

^†^ Community includes teachers, spiritual leaders, general community members, and volunteers. Countries: BDI, Burundi; BEN, Benin; BFA, Burkina Faso; CIV, Côte d'Ivoire; DJI, Djibouti; DRC, Democratic Republic of Congo, ETH, Ethiopia; GHA, Ghana; KEN, Kenya; MWI, Malawi; MOZ, Mozambique; NGA, Nigeria; RWA, Ruwanda; sSUD, south Sudan; TZA, Tanzania; TGO, Togo; UGA, Uganda; ZMB, Zambia; ZWE, Zimbabwe.

Abbreviations: n, number; KAP, Knowledge, attitudes and practices; m, months; HIV, human immunodeficiency virus; TD, truck drivers; FSW, female sex workers; STI, sexually transmitted infections; n/a, not available; ADRA, Adventist Development and Relief Agency; IEC, information, education and communication; PE, peer educator; HCW, healthcare worker; M+Light TD, minibus and light truck drivers; BSS, behavioural surveillance survey; DM, dressmakers; PLWHA, people living with HIV/AIDS; KI, key informant.

Evaluations were conducted between 1990 and 2012 in single countries, with the exception of two programmes that were evaluated across several countries [32, 51]. Programmes tended to be evaluated only once, excluding the Corridors of Hope project that underwent five evaluations, three external and two internal, during the period 2000 to 2009 [27, 40–43]. External evaluators performed five of the 12 evaluations [31, 32, 34, 50, 51]. Methodological approaches and their quality varied across studies from qualitative appraisals, including focus group discussions and interviews, to quantitative designs such as pre- and post-intervention surveys, statistical analysis of routinely-collected data, and trend analysis of repeated cross-sectional surveys. Sampling strategies, when used, and sample sizes also varied across evaluations. Due to the mobility of this population, most of the evaluations had to adjust their sample size and sample method to make use of simple random or purposive sampling. We assessed major limitations in the majority of evaluation studies. These included: 1) absence of control or comparison groups (n = 10),[21, 22, 26, 27, 29, 31, 32, 34, 40–43, 45, 48, 50, 51] hindering the attribution of any effect observed to the programme, and 2) the lack of biological data measurements (e.g. HIV/STI testing), introducing biases in reporting of some of the evaluated outcomes (n = 4) [22, 23, 26, 27, 32, 40–43, 53].

A detailed description of the 75 indicators reported in 12 programme evaluations can be found in Table 3, with the reported results in Table 4. Four programmes reported on inputs and activities available within programmes (i.e. number of sites established, staff trained and resources used) [32, 34, 50, 51]. Only two programmes [50, 51] provided information on costs, however they did not evaluate the programme’s cost-effectiveness. All programmes reported output (n = 42) or outcome indicators (n = 16). Output indicators generally described the volume of services provided and clients reached. Six programmes provided sufficient data on outcome or impact indicators to be included in the meta-analysis. A total of five programmes, evaluated in Benin, Ethiopia, Ghana, Ivory Coast, Kenya, Nigeria, Tanzania, Togo and Zambia, reported one or more impact indicator results [21, 26, 32, 42, 50]. These impact indicators included changes in HIV prevalence for one programme and STI incidence for four [21, 26, 32, 42] programmes. However, only one study conducted biological STI testing. It provided syphilis serology for all patients and further investigation for symptomatic patients, including culture for *Neisseria gonorrhoeae* and *Haemophilus ducreyi*, and antigen detection of *Chlamydia trachomatis* [21]. The other programmes relied on self-reported symptoms. One programme reported changes in AIDS-related mortality, stigma, and social norms using qualitative data [50].

**Table 3:** Mapping of programme results using a programme evaluation framework [39].

Input/Activities		Output		Outcome		Impact	
Indicators	N	Indicators	N	Indicators	N	Indicators	N
***Facilities/services available***	**2**	***HTC services (number of sessions*, *number of patients reached or referred*, *uptake of PICT)***	***5***	***Risk behaviour (condom use*, *number of sexual partners*, *alcohol and drug use)***	***9***	***Health-related***	***5***
n checkpoints per 100 km [32]	1	n HTC sessions and patients receiving HIV test results [34, 40–43, 51]	3	Frequency with sex workers [21, 42]	2	STI incidence [25, 26, 40–43, 45]	4
% sites reporting adequate supply of ABX for STIs [32, 53]	1	n having access to HTC [27, 32, 34, 50, 53]	4	Condoms usage [21, 23, 25–27, 29, 32, 40–43, 45, 48, 50, 53]	9	n AIDS deaths [50]	1
n sites providing HIV-related palliative care [50]	1	% men reporting access to HTC [40–43]	1	Extramarital sex [21]	1	HIV prevalence [53]	1
n sites outlets providing HTC [50, 53]	1	n households referred for HTC [50]	1	Alcohol and drug use [40–43]	1		
n sites accessing QA [50]	1	n individuals receiving PICT [50]	1	n participants using condoms [27]	1		
n targeted condom service outlets [50]	1	n individuals reached through outreach HTC [50]	1	n reported sexual partners [27]	1		
n youth clubs established [50]	1	n pregnant women with known HIV status [51]	1	***Knowledge*, *attitudes*, *and perceptions***	***9***		
% HTC sites supervised [50]	1	***Condoms distribution services***	***4***	Stigma/discrimination towards people with HIV [40–43]	1		
***Staff employed/training provided***	***1***	n condom distributed [23, 32, 34, 53]	3	Risk perception [23, 26, 45]	2		
n trainings conducted [50]	1	n individuals receiving condoms [50]	1	HIV/AIDS awareness and knowledge [26, 29, 31, 32, 42, 45, 48, 50, 53]	6		
n peer educators in the programme [50]	1	***Referral services***	***1***	Perceptions of, and attitudes towards HIV/AIDS [26, 45]	1		
***Materials developed and resources used***	**3**	n HBC programs linked with health centres and hospitals [50]	1	Condom knowledge [22, 48]	2		
n IEC materials developed [50]	1	% HIV-positives referred [50]	1	Knowledge of STIs [40–43]	1		
Average running cost of the clinic [34]	1	***Clients served***	***5***	***Clinical outcomes***	**2**		
Waiting times [34]	1	n satisfied clients [34]	1	% positive among PICT [50]	1		
Budgets/expenditure [51]	1	% target audience reached [50]	1	% positive among HTC [50]	1		
		% reporting exposure to COH [40–43]	1	n having STIs [27]	1		
		Change in clinic attendance [34]	1	% reporting STIs [40–43]	1		
		***Sexual health services***	***2***				
		n visiting for STI management [34]	1				
		n visiting for contraception [34]	1				
		n counselling visits for RH/FP as a result of USG assistance[51]	1				
		***Behaviour change services***	***2***				
		n IEC materials distributed [50]	1				
		n reached with outreach promoting abstinence and/or being faithful [50]	1				
		n reached with outreach promoting behaviour change beyond abstinence and/or being faithful [50]	1				
		n counselled on adherence [50]	1				
		n targeted population reached with individual and/or small group level HIV prevention interventions that are based on evidence and/or meet the minimum standards required [51]	1				
		n MARPS reached with individual and/or small group level HIV prevention interventions that are based on evidence and/or meet the minimum standards required [51]	1				
		n counselling visits for RH/FP as a result of USG assistance [51]	1				
		***Staff trained***	***1***				
		n trained in programmes promoting abstinence and/or being faithful [50]	1				
		n trained in programmes promoting behaviour change beyond abstinence and/or being faithful [50]	1				
		n individuals trained to provide HIV related palliative care [50]	1				
		n individuals trained in HTC [50, 53]	1				
		n trained in FP/RH with USG funds [51]	1				
		***Other services***	***2***				
		n provided with HIV-related palliative care [50]	1				
		n screened for TB symptoms [50]	1				
		n on cotrimoxazole prophylaxis [50]	1				
		n on food and nutrition support [50]	1				
		n receiving insecticide-treated bed nets [50]	1				
		n receiving safe water treatment [50]	1				
		n circumcised [42]	1				
		Change in ART access [50]	1				
		n people who have seen or heard a specific USG supported FP/RH message [51]	1				
		n PLHIV reached with a minimum package of PwP interventions [51]	1				
		n eligible adults and children provided with a minimum of one care service [51]	1				
		n eligible clients who received at least 1 PLHIV care and support service [51]	1				

Abbreviations: n, number; prog, programme; HTC, HIV testing and counselling; PICT, provider-initiated counselling and testing; STI, sexually transmitted infections; AIDS, acquired immunodeficiency syndrome; HIV, human immunodeficiency virus; ABX, antibiotics; QA, quality assurance; %, percentage; HBC, home-based care; IEC, information, education and communication; COH, corridors of hope; TB, tuberculosis; ART, antiretroviral treatment; PwP, prevention with positives; PLHIV, people living with HIV.

**Table 4:** Evaluation results of programmes reporting changes in indicator results for TD populations only.

Indicators	Programme	Country	Reported change
***Output indicators***			
Change in HTC uptake and receiving test results	Corridors of Hope [42]	ZMB	Increase (2000–2009) in TD who had reported ever doing HTC: from 33·5–49· 4 Increase (2000–2009) in TD who received HTC results: from 90·4 to 98·9%
	Reproductive Health services for populations at high risk of HIV in Mozambique [34]	MOZ	Increase (2004–2009) in average number of tests performed observed in all populations: from 54·0 to 115·0 per month
Change in HTC access	High Risk Corridor Initiative [53]	ETH	Increase (2005–2007) in number of people who used HTC centres along the corridor: from 1,000 to 27,639
			Increase (2005–2007) in number of border crossing points with HTC centres: from 3 to 16
Change in condom distribution volume	Prevention of sexual transmission of HIV through IEC [25]	MWI	Relative increase compared to baseline (1996): 33·0% in active PE arm, 2·5% in no PE arm, 8·5% in average arm (HCW visits but no PE)
	Reproductive Health services for populations at high risk of HIV in Mozambique [34]	MOZ	Increase (2004–2009) in total volume for the period from 3,151 to 9,200
	High Risk Corridor Initiative [53]	ETH	Increase (2005–2007) in condoms distributed throughout the corridor: from 0·97 million to 8·8 million
Change in ART access	High Risk Corridor Initiative [50]	ETH	Increase (2005–2007) in number of people accessing ART post HTC or HBC referrals (no reported statistics—narrative)
Change in clinic attendance	Reproductive Health services for populations at high risk of HIV in Mozambique [34]	MOZ	Increase in all clinic visit (2004–2009) from 206 to 475 per month; in STI visits from 20 to 28
***Outcome indicators***			
Change in sexual partners	Corridors of Hope [42]	ZMB	*Increase in protective sexual behaviours* (2000–2009) Did not have sex with a regular SP in last 12m: from 0.4% to 43·0%; reported not having sex with FSW in last 12m: from 68·0% to 78·0%; reported not having sex with a non-regular, non-commercial SP in last 12m: from 73·0% to 99·0% *Decrease in risk sexual behaviours* (2000–2009) Reported two or more regular SP in last 12m: from 21·0% to 4·0%; reported two or more commercial SP in last 12m, from 22·0% to 15%; reported sex with two or more non-regular, commercial SP in last 12m: from 8·0% to 1·0%
	Behavioural risk-reduction programme [21]	KEN	Decrease in % reporting extramarital sex (1993–1994): from 49% to 36%
Change in condom use	Corridors of Hope [42]	ZMB	(2000–2009) Reported using a condom during last sex with a SW: from 93% to 97%; reported consistent condom use with SW: from 84% to 91%; reported condom use on last occasion with a regular SP in last 12 months: from 43·0% to 73·0%; reported consistent condom use with regular SP: from 8·0% to 60·0%
	NECTOI's AIDS education programme [29]	ZWE	Increase (1995–1997) in % reporting consistent condom use: from 72·0% to 82·0%. (SW: Increase (1995–1997) in consistent condom use: from 82·2% to 88·6%)
	AMREF [26, 45]	TZA	Increase (1990–1991) in ever using condoms: from 56·1% to 73·7%. Decrease (1991–1993) in ever using condoms: from 73·7% to 71·5%.
	The HIV/AIDS project for the Abidjan-Lagos Corridor* [32, 53]	BEN, GHA, CIV, NGA, TGO	Increase (2005–2007) in reported condom use during last sex act with non-regular SP in last 12 months: from 59·0% to 78·8% (among SW: from 58·8% to 70·5%)
	Prevention of sexual transmission of HIV through IEC [25]	MWI	(Increase (1996) in having ever used condoms among SW: from 66·5% to 100·0%)
	Behavioural risk-reduction programme [21]	KEN	Decrease (1993–1994) in consistent condom use during extramarital sex: from 34·0% to 29·0%
	Prévention du SIDA sur les Axes Migratoire de l’Afrique de l’Ouest (PSAMAO) [29]	BFA	Increase (1997–2000) in condom use with occasional sexual partners: no reported statistics—narrative
	Improving HIV/AIDS knowledge and risk behaviours of drivers in Nigeria [48]	NGA	Increase (2011) in current condom use: from 34·3% to 51·9%
	High Risk Corridor Initiative [50]	ETH	Increase (2005–2007) in condom use: no reported statistics—narrative
Change in alcohol and drug use	Corridors of Hope [42]	ZMB	Decrease (2000–2009) in alcohol and drug use: from 11·0% to 3·0%
Change in HIV/AIDS awareness and knowledge	ADRA's HIV/AIDS Programme [31]	GHA	Increase (2006) in knowledge about difference between HIV and AIDS; transmission modes and signs and symptoms of HIV infected persons: no reported statistics—narrative
	AMREF [26, 45]	TZA	Increase (1991–1993) in % reporting accurate knowledge about intercourse as mode of transmission: from 97·4% to 99%. (SW: from 93·4% to 97·2%) Decrease (1991–1993) in % reporting misconceptions about modes of transmission: shaking hands, from 40·7% to 17·4% (SW: from 42·4% to 19·2%); mosquitos, from 55·5% to 43·3% (SW: 61·8% to 45·3%)
	Corridors of Hope [42]	ZMB	Increase (2000–2009) in % TD who know that abstinence can prevent HIV: Livingstone, from 93·0% to 99·3%; Chirundu, from 88·2% to 97·9%; Kapiri Moshi, from 92·1% to 98·6%. Decrease (2000–2009) in % TD who think that HIV can be transmitted by mosquitos: Livingstone, from 15·8% to 7·3%; Chirundu, from 17·6% to 7·8%; Kapiri Moshi, from 26·1% to 9·1%. Change (2000–2009) in % TD who think that HIV can be transmitted through sharing a meal: Livingstone, from 5·5% to 4·0%; Chirundu, from 9·0% to 10·3%; Kapiri Moshi, from 23·1% to 12·9%
	High Risk Corridor Initiative [50]	ETH	Increase (2005–2007) in TD being able to identify at least two ways to prevent HIV/AIDS: from 68·0% to 82·7%. (Increase (2005–2007) in SW being able to identify at least two ways to prevent HIV/AIDS: from 59·5% to 87·9%)
	Improving HIV/AIDS knowledge and risk behaviours of drivers in Nigeria [48]	NGA	Increase (2011) in knowledge about HIV/AIDS: from 89·3% to 100·0%
	Prévention du SIDA sur les Axes Migratoire de l’Afrique de l’Ouest (PSAMAO) [29]	BFA	Increase (1997–2000) in HIV/AIDS knowledge: no reported statistics—narrative
	The HIV/AIDS project for the Abidjan-Lagos Corridor* [32, 50, 53]	BEN, GHA, CIV, NGA, TGO	Increase (2005–2007) in TD identifying at least two ways to prevent HIV: from 68·0% to 90·0% (SW: from 59·5% to 90%)
Change in risk perception	AMREF [26, 45]	TZA	Increase (1991–1993) in perceiving self at risk: from 54·5% to 62·1% Increase (1991–1993) % reporting positive attitudes: willing to live in the same house, from 66·7% to 90·8% (SW: from 43·3% to 79·9%); willing to eat together, from 57·6% to 69·5% (SW: 33·5% to 52·5%); willing to share toilet, from 53·1% to 56·1% (SW: from 81·9% to 97·2%)
	NECTOI's AIDS education programme [22]	ZWE	Decrease (1995–1997) in % perceiving self at risk: from 52·7% to 30·2% (SW: from 44·6% to 13·2%)
Change condom knowledge	Improving HIV/AIDS knowledge and risk behaviours of drivers in Nigeria [48]	NGA	Increase (2011) in awareness of condom use as a preventive practice: from 81·4% to 100·0%
	NECTOI's AIDS education programme [22]	ZWE	Increase (1995–1997) in HIV awareness and condom use: no reported statistics—narrative
Change in knowledge of STIs	Corridors of Hope [40, 42]	ZMB	Increase (2006–2009) in % knowing two or more STI symptoms: in men, from 73.0% to 81·2% and in women, 44·0% to 59·6%
***Impact indicators***			
Change in STI incidence	Corridors of Hope [42]	ZMB	Decrease in proportion reporting genital discharge in the last 12m: from 6·4 (2000) to 5·7 (2003) to 4·9 (2006) to 3·4% (2009); genital ulcers/sores in the last 12m: from 5·3 (2000) to 8 (2003) to 2·8 (2006) to 2·2% (2009)
	AMREF [26]	TZA	Increase in proportion of TD reporting ever having had an STI: from 40·2% to 56·7%. (SW: from 15·5% to 36·8%)
	The HIV/AIDS project for the Abidjan-Lagos Corridor* [32, 53]	BEN, GHA, CIV, NGA, TGO	Increase in reported STI incidence: from 6·7 to 11·5/100 person-yrs. (Decrease in STI prevalence among SW: from 8·9% to 3·8%)
	Behavioural risk-reduction programme [21]	KEN	Decrease in STI incidence: from 34·0 to 10/100 person-yrs
Change in HIV prevalence	The HIV/AIDS project for the Abidjan-Lagos Corridor* [32, 53]	BEN, GHA, CIV, NGA, TGO	Decrease in HIV prevalence: from 5.0% (Feb 2005)/2.7% (Dec 2005) to 1.7% (2007). (Uncertain trend in HIV prevalence among FSW: from 30.1% (Feb 2005)/12.7% (Dec 2005) to 20.7% (2007)).
Change in AIDS deaths	High Risk Corridor Initiative [50]	ETH	Decrease in AIDS deaths among all MARP from routinely-collected data. Only narrative description

* Currently: Organisation du Corridor Abidjan-Lagos (OCAL) project. Countries: BEN, Benin; BFA, Burkina Faso; CIV, Côte d'Ivoire; ETH, Ethiopia; GHA, Ghana; KEN, Kenya; MWI, Malawi; MOZ, Mozambique; NGA, Nigeria; TZA, Tanzania; TGO, Togo; ZMB, Zambia; ZWE, Zimbabwe. Abbreviations: HTC, HIV testing and counselling; HIV, human immunodeficiency virus; IEC, information education communication; PE, peer educator; HCW, healthcare worker; ART, antiretroviral treatment; STI, sexually transmitted infection; SP, sexual partner; m, months; (F)SW, (female) sex worker; NECTOI, National Employment Council for the Transport Operating Industry; AMREF, African Medical & Research Foundation; AIDS, acquired immunodeficiency syndrome; STD, sexually transmitted disease; ADRA, Adventist Development and Relief Agency; yrs, years; MARP, most-at-risk-populations, FGC, female genital cutting.

### Impact of healthcare programmes

**Fig 3:**
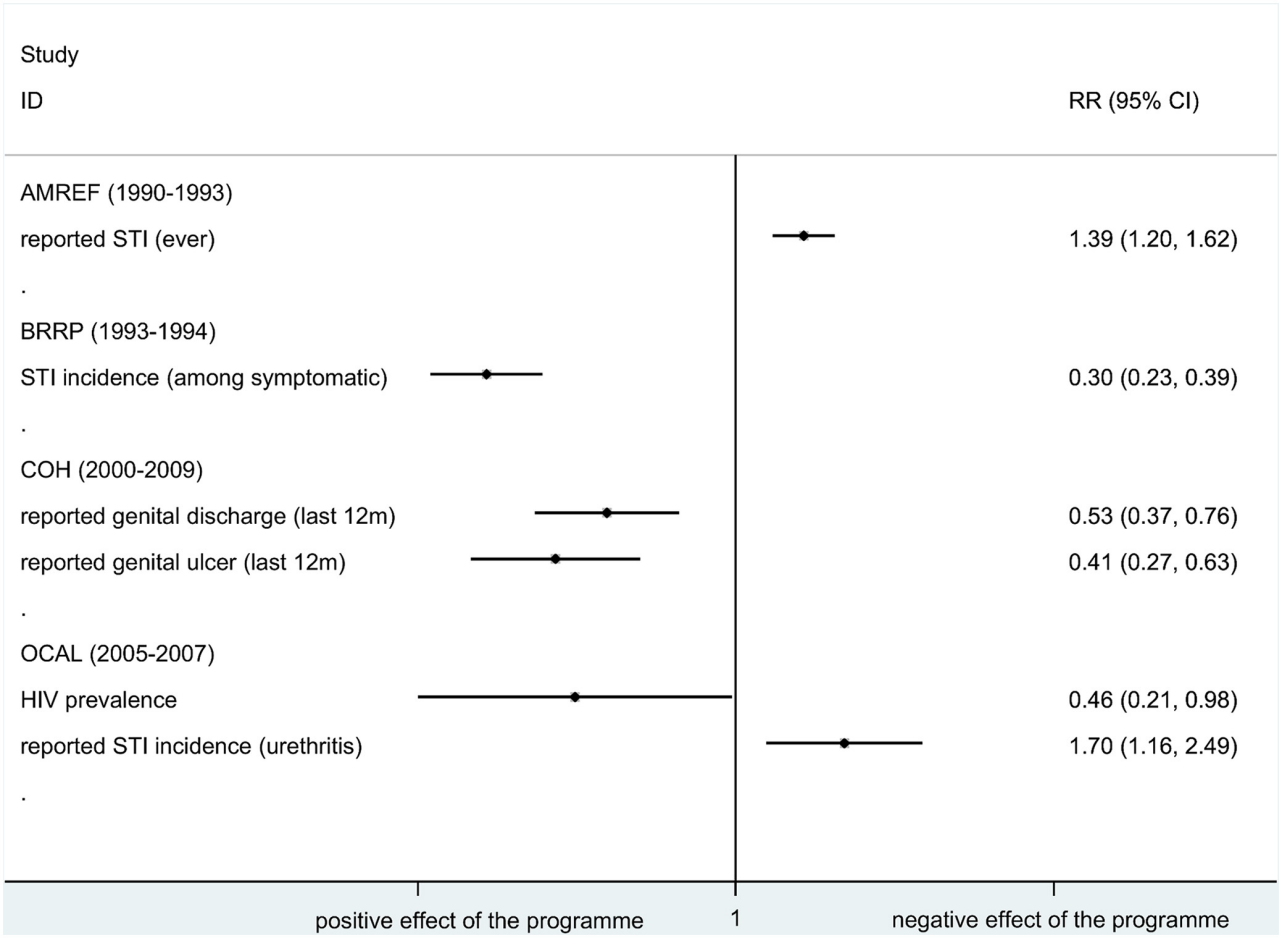
Relative risk in impact indicators by programme. ID, identification; RR, relative risk; 95% CI, 95% confidence interval; HIV, human immunodeficiency virus; STI, sexually transmitted infection; OCAL, The HIV/AIDS project for the Abidjan-Lagos Corridor, currently Organisation du Corridor Abidjan-Lagos project; AMREF, African Medical & Research Foundation; BRRP, Behavioural risk-reduction programme; COH, Corridors of Hope.

**Fig 4:**
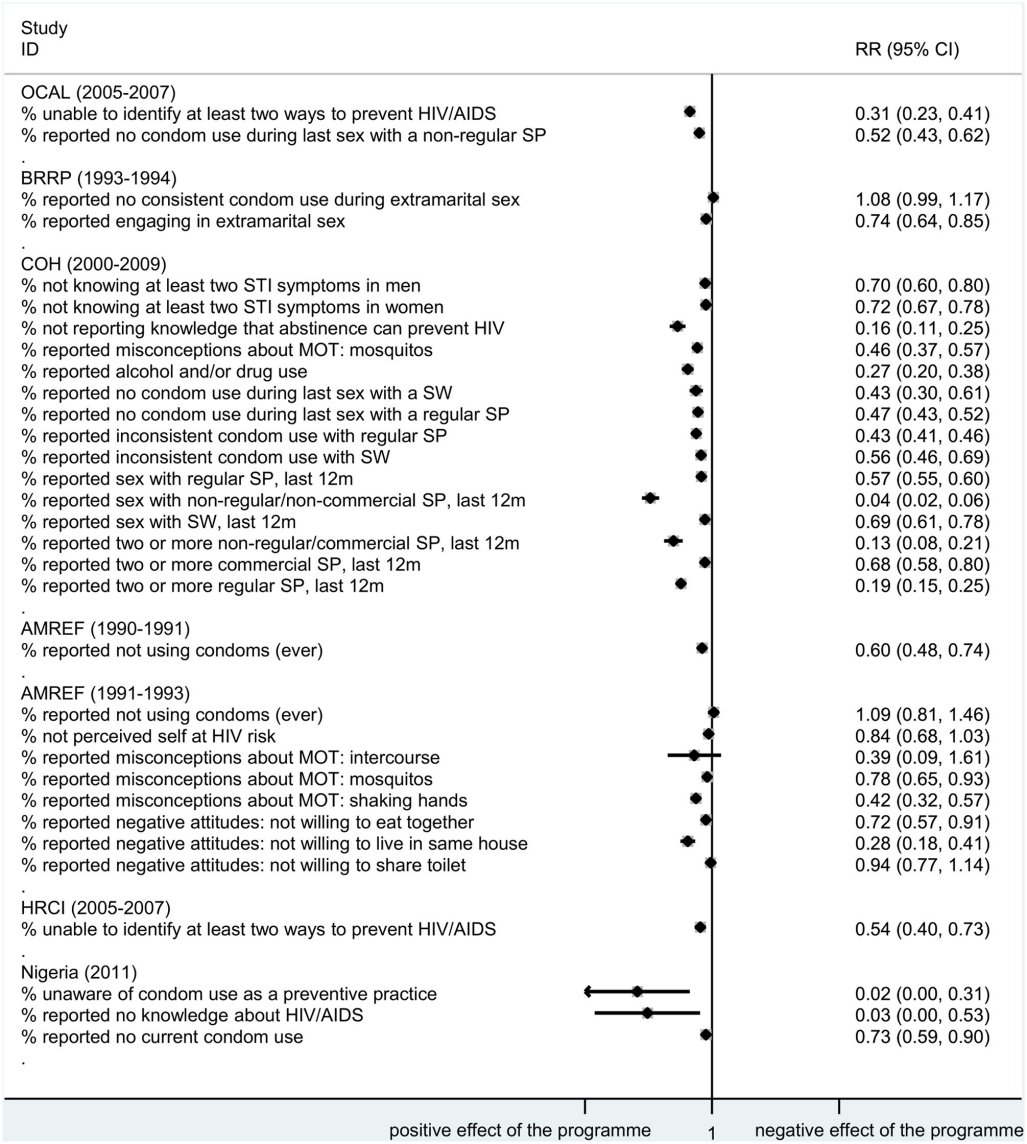
Relative risk in outcome indicators by programme. ID, identification; RR, relative risk; 95% CI, 95% confidence interval; HIV, human immunodeficiency virus; STI, sexually transmitted infection; OCAL, The HIV/AIDS project for the Abidjan-Lagos Corridor, currently Organisation du Corridor Abidjan-Lagos project; AMREF, African Medical & Research Foundation; BRRP, Behavioural risk-reduction programme; COH, Corridors of Hope; HRCI, High Risk Corridor Initiative; Nigeria, Improving HIV/AIDS knowledge and risk behaviours of drivers; m, months; SP, sexual partner; MOT, modes of transmission; SW, sex worker; AIDS, acquired immunodeficiency syndrome.

In Figs 3 and 4 we provide an overview of the potential impact on truck driver health by programme by plotting the RRs and 95% CI for all reported outcomes and impact indicators.

The programmes reported mixed results on impact indicators with two [26, 32, 53] out of four programmes [21, 26, 32, 42, 53] showing an increase in reported prevalence of STIs. Results for outcome indicators were consistent across the programmes, with decreases in reported risk behaviours or misconceptions and negative attitudes towards people living with HIV. All programmes reported substantial increases in output indicators, such as the number of HTC sessions or the numbers of condoms distributed (Table 4).

**Fig 5:**
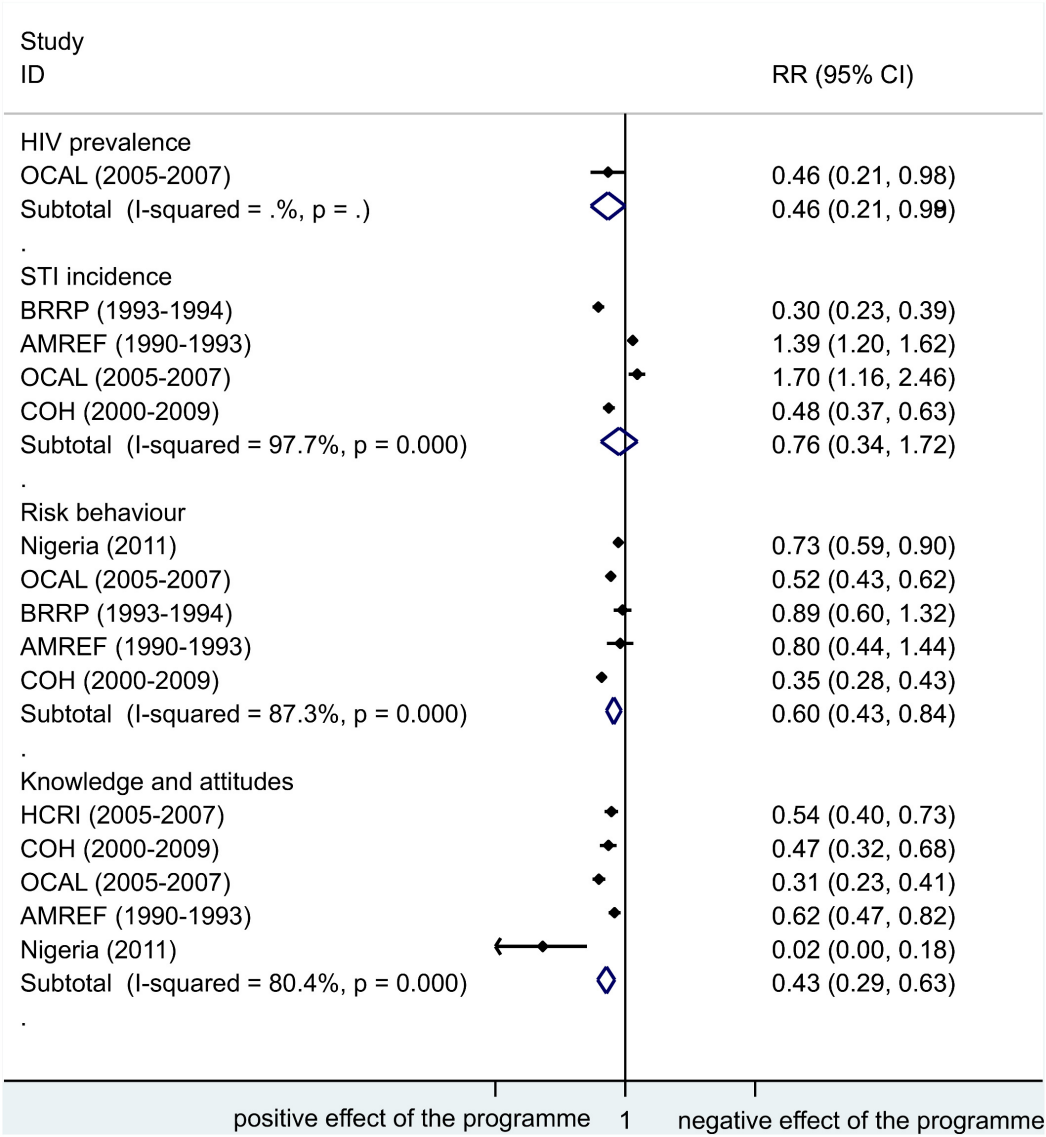
Subgroup analysis, summary relative risk estimates by type of indicator. ID, identification; RR, relative risk; 95% CI, 95% confidence interval; HIV, human immunodeficiency virus; OCAL, The HIV/AIDS project for the Abidjan-Lagos Corridor, currently Organisation du Corridor Abidjan-Lagos project; STI, sexually transmitted infection; BRRP, Behavioural risk-reduction programme; AMREF, African Medical & Research Foundation; COH, Corridors of Hope; Nigeria, Improving HIV/AIDS knowledge and risk behaviours of drivers; HRCI, High Risk Corridor Initiative.

Finally, in Fig 5 we present a subgroup analysis exploring the heterogeneity in the results by type of indicator reported. While the indicators reported did not explain all the heterogeneity present in the results (with all p values <0.001 for all subgroups analysed), the main impact of these programmes can be shown as a significant reduction in risk behaviours and negative attitudes and misconceptions reported against people living with HIV, with all RRs below one. Alternative S1, S2 and S3 Figs are presented in the supplementary material plotting risk differences and 95% CI for all outcomes and impact indicators and using a fixed-effects model as an alternative to the random effects model, respectively. Our results were robust to these sensitivity analyses.

## Discussion

In this systematic review we describe 22 healthcare programmes prioritising truck drivers that have been implemented in 30 countries across sub-Saharan Africa. These healthcare programmes included short-term interventions of narrow scope, such as limited primary health care and diagnosis of communicable diseases. A minority of healthcare programmes expanded the scope to include gender based violence reduction and palliative care. While most countries had or have healthcare programmes for truck drivers that provide services of HIV prevention and diagnosis, no antiretroviral treatment (ART) services have been offered on site. This provision of vertical services reflects the overall regional HIV disease burden and the particular focus of donors, while the lack of ART provision of reflects the logistical challenges of treatment provision across borders. Regionally, HIV prevalence estimates among truck drivers have been reported as high. Studies from Nigeria show a 10% HIV prevalence among truck drivers [54] while South African surveys have reported HIV prevalence estimates in truck drivers who were clients of female sex workers as high as 56% [55] and in truck drivers in general as 26% [56], in 2001 and 2003/4 respectively [57]. This increased vulnerability to acquiring and transmitting HIV among truck drivers has prompted UNAIDS and national governments to identify them as a key population, prioritising this group in their HIV programmes, as does South Africa’s National Strategic Plan on HIV, TB and STIs 2012–2016 [11], for example. However, the majority of the programmes in this review did not actively seek to identify people living with HIV (through HTC) and link them to care, but rather focussed on healthcare messaging and BCC. Although this is in keeping with findings of the 2012 review of sex worker programmes in Africa [58], healthcare programmes for truck drivers have seen a progression in scope from 2001 onwards reflected in the increasing (though still insufficient) number of services delivered from satellite and mobile clinics. The World Health Organisation and UNAIDS focus on increasing testing and linkage to ART care can be translated, among truck drivers, into 90% of truck drivers living with HIV being aware of their HIV-positive status, 90% of HIV-positive truck drivers being on ART, and 90% of truck drivers on ART achieving viral suppression [59]. In the absence of data, and with the very limited coverage of these programs, it seems that there is a considerable way to go from what appears to be a low baseline for all three of these indicators in truck-driver populations in sub-Saharan Africa.

We also observe that, in terms of evaluations, just above half of these programmes have been evaluated to date and, of these, most looked only at input or outcome indicators. The evaluation methodologies relied on routinely-collected data and were constrained by low evaluation budgets. In planning for scale-up of programmes, Ministries of Health need to know which interventions, delivered in which way, and at what cost were more effective and efficient. Rigorous measurement of programme outcomes and impact is therefore needed to be able to support wider implementation of programmes. Yet, only six programmes reported sufficient data to analyse outcome or impact indicators. Moreover, programmes prioritising truck drivers have failed to demonstrate an attributable impact on STI incidence or HIV prevalence, due to study designs that lack control arms and heterogeneous trends in HIV risk reduction. However, the programmes overall could be considered to have had a positive effect on risk behaviours, knowledge, and attitudes, albeit the issue of attribution also remains open for these outcomes. For continuous improvement of programme delivery, monitoring and evaluation (M&E) needs to be built into all programmes at the design stage [60]. In the absence of good M&E frameworks, these programmes lose out on valuable process information and are less able to identify and address gaps and make informed decisions regarding operations management and service delivery, including effective and efficient use of resources.

The majority of the programmes were funded by agencies external to the country of implementation and for limited periods of time. Only three programs were funded by trucking companies/bodies. This funding situation influences the evaluation aim and objectives. Firstly, the need to report specific indicators for programme monitoring and reporting is donor-dictated, influencing the evaluation design and generally streamlining data collection to be compliant with donor priorities. Since main indicators are input, output, and outcome monitoring data, programmes remain deficient in measures to evaluate impact unless a separate study is envisaged. Secondly, programmes that are proven efficient and have an impact should be scaled-up. Yet scalability and sustainability are real concerns where implementing countries and relevant stakeholders are not closely involved and have no ownership of programmes.

Our study presents several limitations. First, we aimed to describe healthcare programmes prioritising truck drivers based on information available. Due to the nature of the documents reviewed, we were unable to assess the quality of services provided in these programmes and we limited our quality assessment to the methodologies employed where an evaluation was conducted. Additionally, we focused on reports and programme descriptions available publicly. This might have limited the completeness of our mapping. However, we contacted stakeholders and efforts were made to include all grey literature. We performed a meta-analysis to summarise the results of programme evaluations. We aimed therefore to quantify the impact these programmes have had on truck driver health indicators. However, due to the diversity of indicators reported, we were able to use the meta-analysis results to make qualitative statements as to where the programmes had a positive impact. Finally, we found an important heterogeneity in the results presented. We explored this heterogeneity in a subgroup analysis by type of indicator reported. Other covariates that could help explain the heterogeneity observed include the study setting, study type or even the choice of measurement. Due to the limited number of studies in each of these sub categories, we were unable to produce subgroup analyses for all covariates or to run a meta-regression to quantify their impact on the variance.

## Conclusion

Diverse healthcare programmes prioritising truck drivers have been implemented in 30 sub-Saharan African countries since 1989. Just over half of these healthcare programmes have been evaluated. Among those evaluated, potential benefits to truck drivers have been shown. However, information gaps about availability of services and their effects impede further planning and implementation of effective healthcare programmes for truck drivers.

Collaborative efforts among workplaces, governments, and trucking governing bodies are essential to the design of effective programmes. Given the mobility associated with this population’s occupation, inter-governmental collaboration is imperative to facilitate service delivery along the trucking corridors and ensure continuity of care. The interconnected nature of the transportation network provides a unique opportunity that should be taken advantage of to establish stronger linkages to healthcare programmes and provision of services for this important population. Without this, truck drivers will be left behind in the move to achieve the global targets for access and linkage to care in sub-Saharan Africa.

## Supporting Information

S1 Fig Risk differences in impact indicators by programme. (TIF)

S2 Fig Risk differences in outcome indicators by programme. (TIF)

S3 Fig Subgroup analysis, summary relative risk estimates by type of indicator (fixed effects model). (TIF)

S1 Table PRISMA Checklist. (PDF)
