# Supplementary figures and images for: Healthcare Programmes for Truck Drivers in Sub-Saharan Africa: A Systematic Review and Meta-Analysis
Source: PLoS One. 2016 Jun 22;11(6):e0156975. doi: 10.1371/journal.pone.0156975 (PMC4917167; doi:10.1371/journal.pone.0156975)

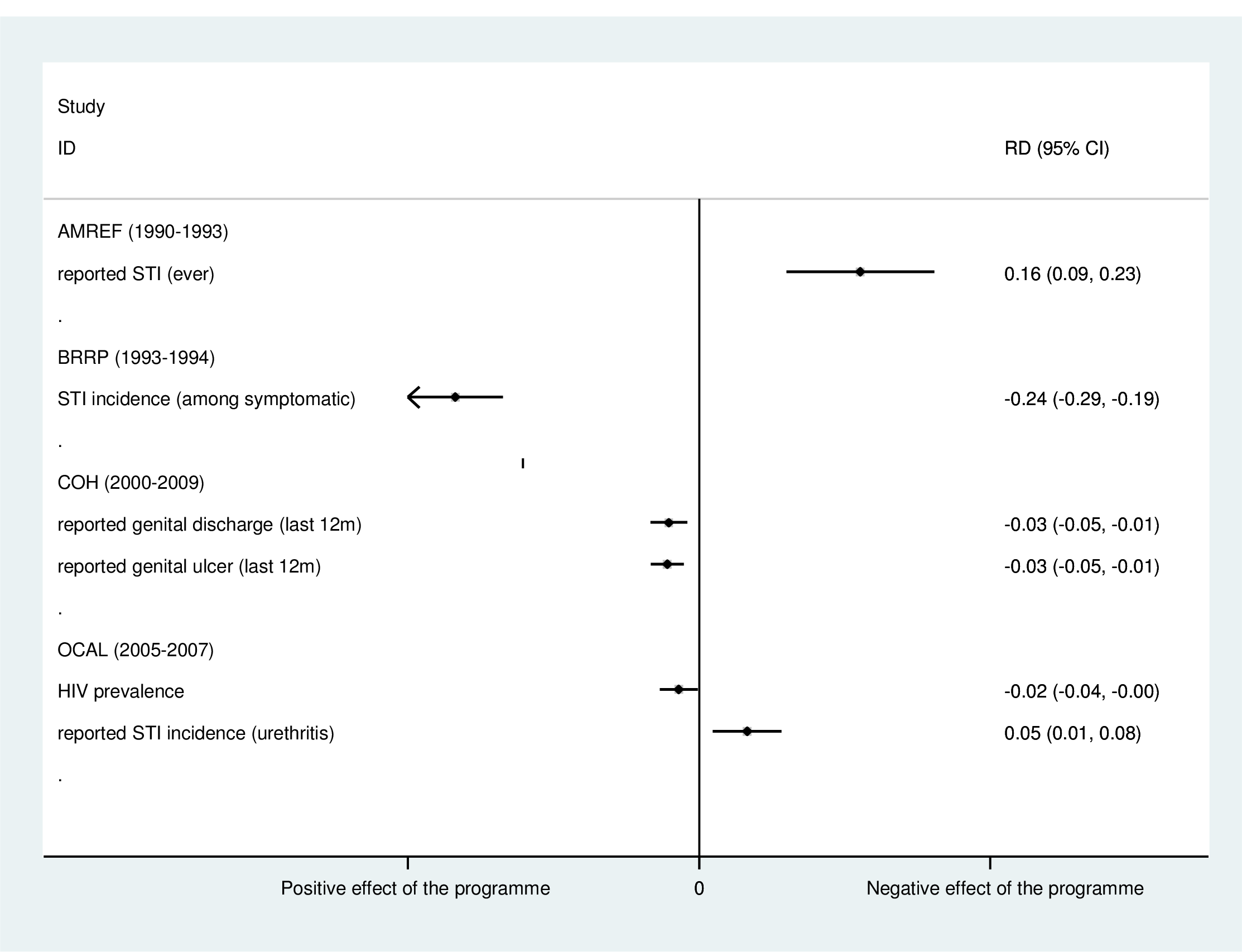

Supplement: S1 Fig — (TIF) [file pone.0156975.s001.tif]

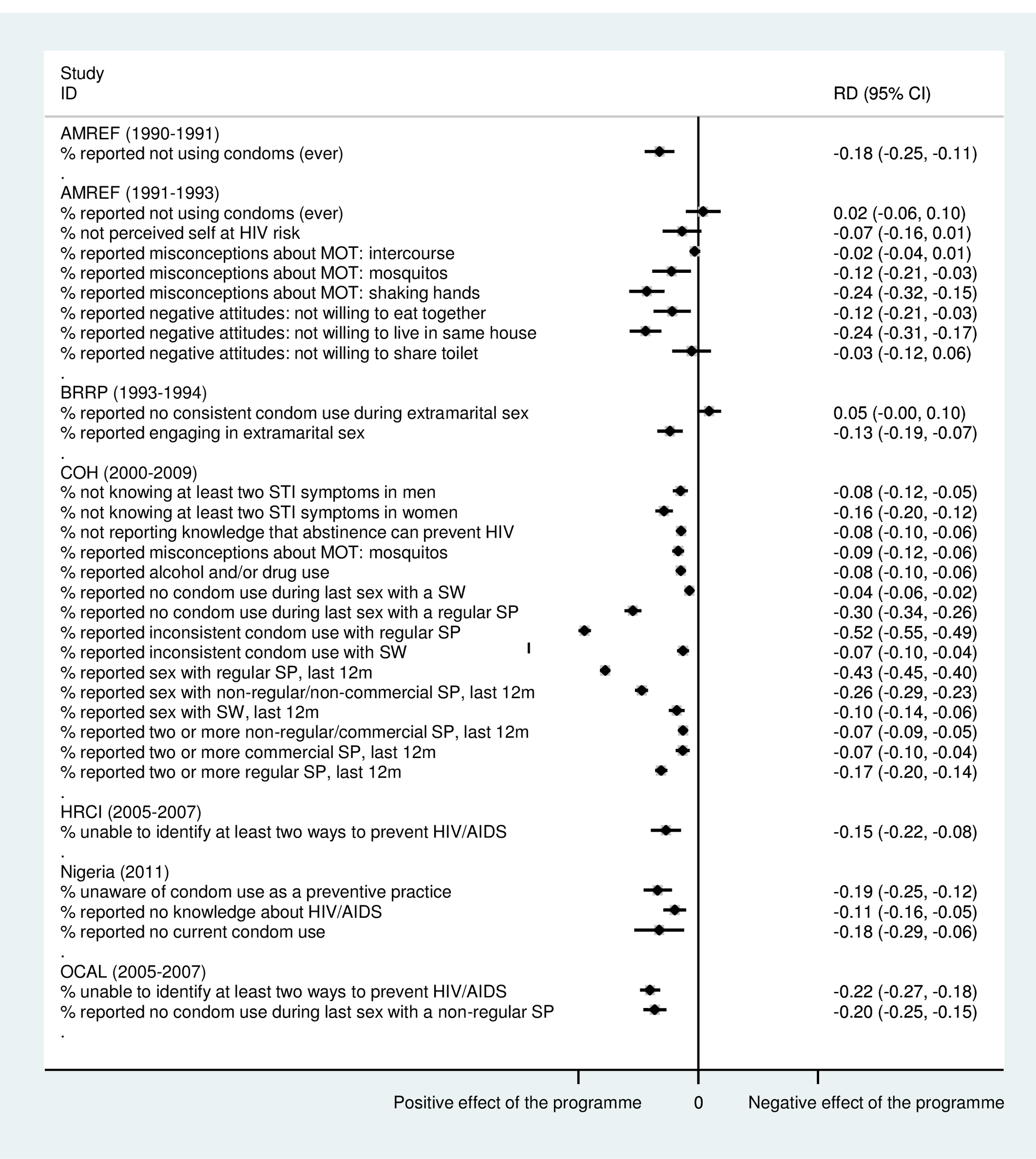

Supplement: S2 Fig — (TIF) [file pone.0156975.s002.tif]

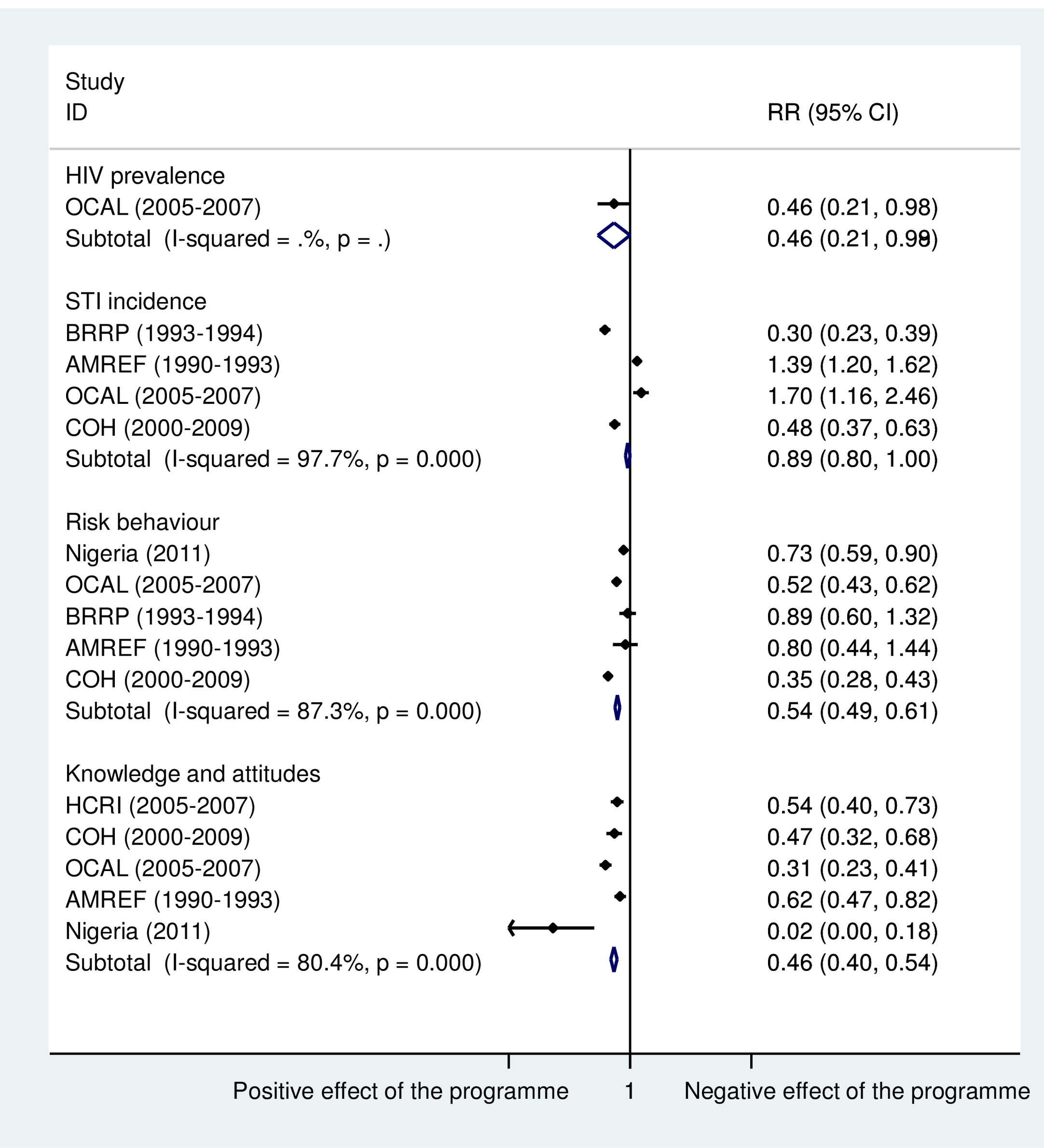

Supplement: S3 Fig — (TIF) [file pone.0156975.s003.tif]
